# Supplementary material for: Overexpression profiling reveals cellular requirements in the context of genetic backgrounds and environments
Source: PLoS Genet. 2023 Apr 28;19(4):e1010732. doi: 10.1371/journal.pgen.1010732 (PMC10171610; doi:10.1371/journal.pgen.1010732)
Supplement: S1 Fig — (PDF) [file pgen.1010732.s001.pdf]

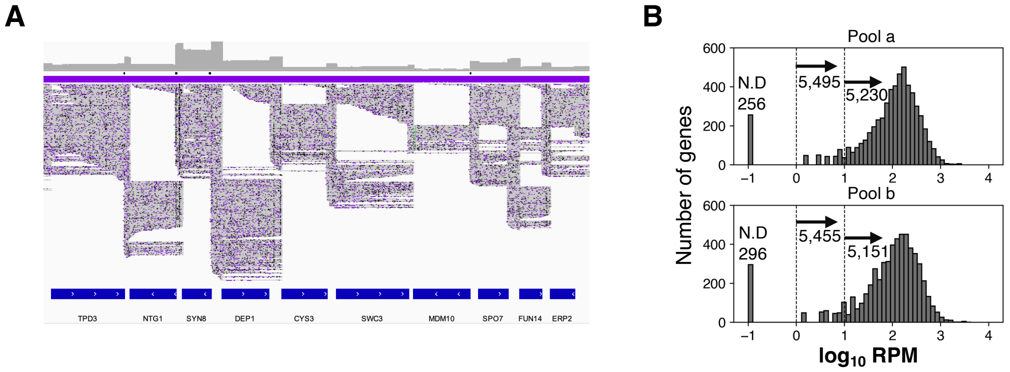

S1 Fig. Coverage of pooled libraries.

(**A**) An example of plasmid inserts sequencing results visualized using a genome browser, IGV [1]. (**B)** Distribution of plasmid inserts (RPM) in Pool_a (top) and Pool_b (bottom). For convenience, those with an RPM of 0 have been appended with 0.1. Vertical dashed lines indicate RPM ≥1 or RPM ≥10. The mean, median, and standard deviation of RPM in Pool_a were 76.8, 120, and 6.42, respectively. In Pool_b, these values were 70.6, 115, and 7.05.

Reference

1. Robinson JT, Thorvaldsdóttir H, Winckler W, Guttman M, Lander ES, Getz G, et al. Integrative genomics viewer. Nat Biotechnol. 2011;29: 24–26.
