# Supplementary material for: Overexpression profiling reveals cellular requirements in the context of genetic backgrounds and environments
Source: PLoS Genet. 2023 Apr 28;19(4):e1010732. doi: 10.1371/journal.pgen.1010732 (PMC10171610; doi:10.1371/journal.pgen.1010732)
Supplement: S2 Fig — (PDF) [file pgen.1010732.s002.pdf]

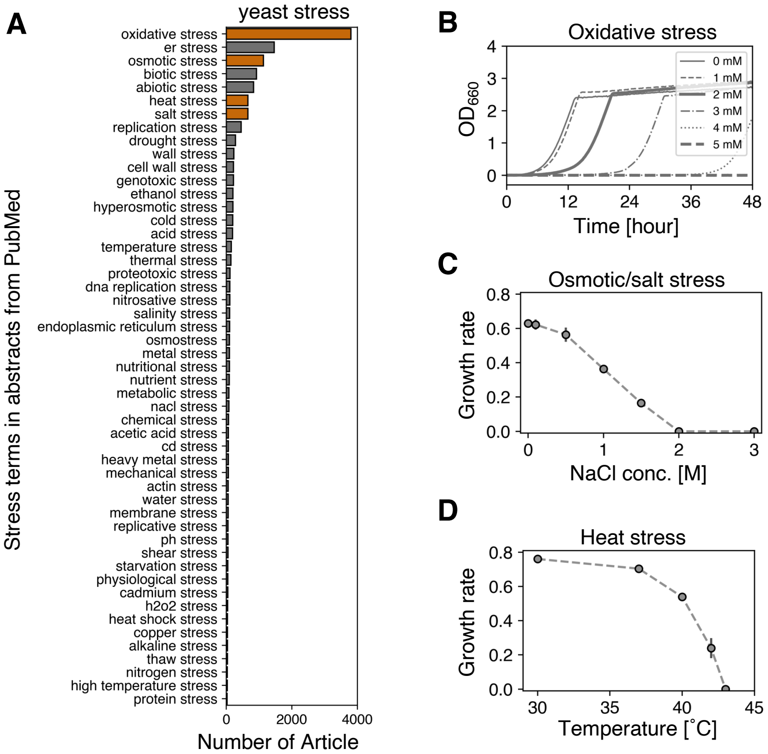

S2 Fig. Growth of yeast cells under three most-studied stresses.

(**A**) Number of articles in which "XXX stress" appears in the abstract of articles obtained from a PubMed search for "yeast stress". The orange bars indicate the stresses that were focused on in this study. (**B)** Growth curves of BY4741 with the indicated amounts of H_2O_2 in YPD. (**C**) Growth rates of BY4741 with the indicated amounts of NaCl in YPD. (**D**) Growth rates of BY4741 in YPD under the indicated temperatures.
