# Supplementary material for: Overexpression profiling reveals cellular requirements in the context of genetic backgrounds and environments
Source: PLoS Genet. 2023 Apr 28;19(4):e1010732. doi: 10.1371/journal.pgen.1010732 (PMC10171610; doi:10.1371/journal.pgen.1010732)
Supplement: S3 Fig — (PDF) [file pgen.1010732.s003.pdf]

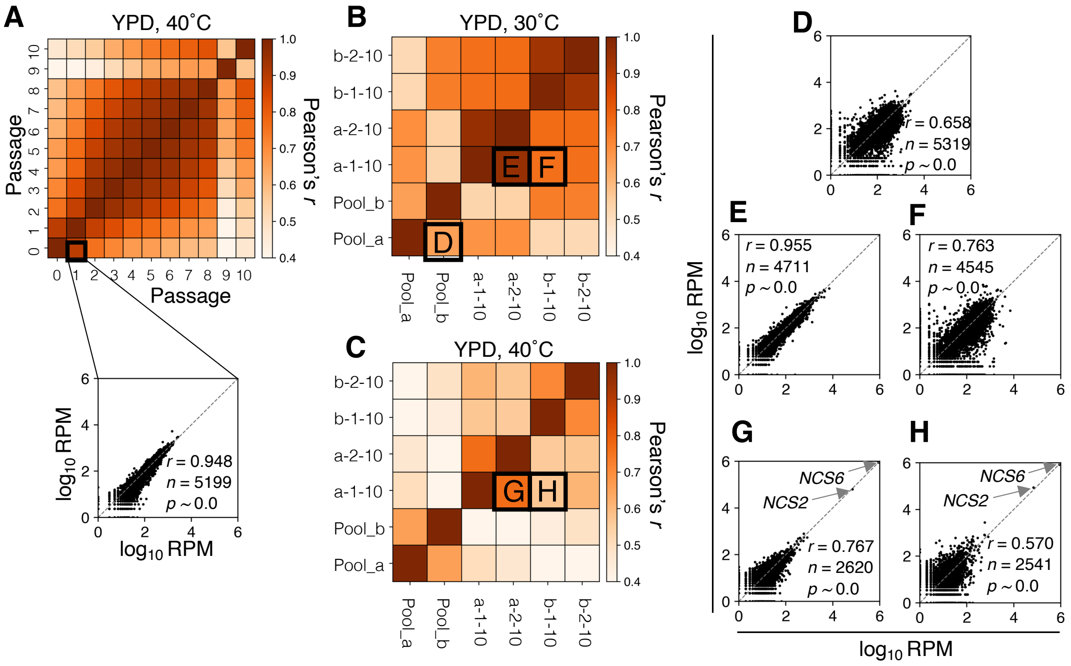

S3 Fig. Reproducibility of overexpression profiling.

(**A**) A heatmap showing Pearson's correlation among each passage in the identical replicate (Pool_a-1) under 40°C. A purple-to-orange color scale represents low to high Pearson's correlations. The lower panel shows a scatter plot comparing RPM before and after the 1st passage. (**B-C**) RPM scores were quite reproducible between the replicates that originated from the identical pool, while they had some differences when the originating pools were different. Heatmaps showing Pearson's correlation among four replicates on the 10th passages under (**B**) 30°C and (**C**) 40°C. "a-" and "b-" in the four replicates originated from Pool_a and Pool_b respectively. The comparisons **D-H** on graphs **B** and **C** are shown as independent scatter plots in **D-H**. (**D-H**) Scatter plots comparing RPM. The comparisons are described in **B** and **C**. "*r*", "*n*", and "*p*" mean correlation coefficients, sample number, and p-values, respectively. "$p \sim 0$" indicates that the *p*-value is smaller than the value that can be calculated.
