# Supplementary material for: Overexpression profiling reveals cellular requirements in the context of genetic backgrounds and environments
Source: PLoS Genet. 2023 Apr 28;19(4):e1010732. doi: 10.1371/journal.pgen.1010732 (PMC10171610; doi:10.1371/journal.pgen.1010732)
Supplement: S4 Fig — (PDF) [file pgen.1010732.s004.pdf]

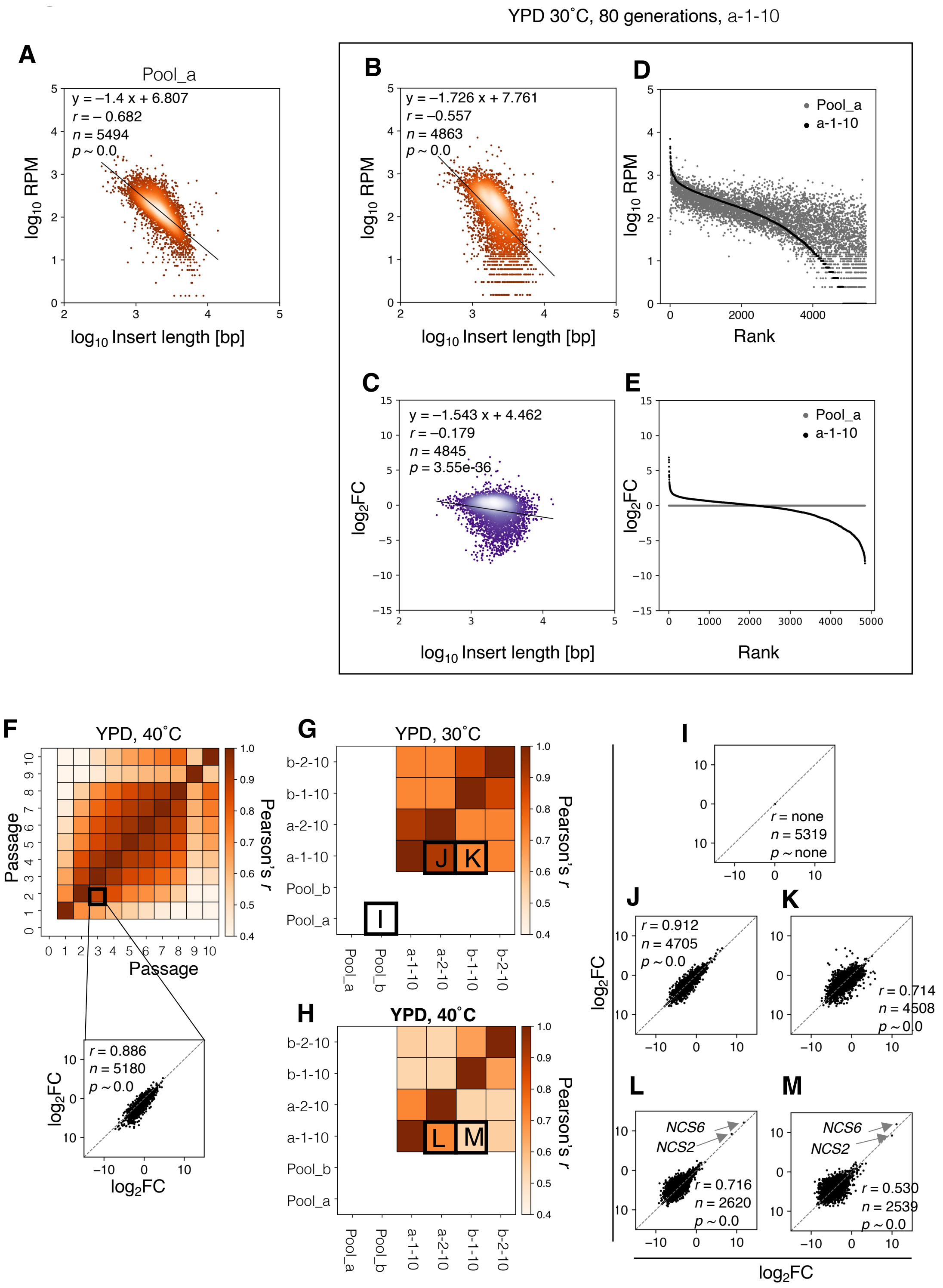

S4 Fig. Correction of frequency bias by insert length.

(**A-B**) Negative correlations between insert frequency (RPM) and insert length (bp) in the initial (**A**) and 10th passage (**B**) of Pool_a. The solid lines indicate regression lines (regression equations are shown in the figure). The shading of colors implies the density

of the points, with thinner lines indicating higher density. (**C**) A scatter plot comparing fold change (FC) and insert length in the 10th passage. The solid lines indicate regression lines (regression equations are shown in the figure). The shading of colors implies the density of the points, with thinner lines indicating higher density. (**D-E**) Scatter plots showing RPM (**E**) or fold change (**F**) in the initial pool (grey) and the 10th passage (dark grey). These scores were sorted in descending order of RPM and fold change in the 10th passage. (**F**) A heatmap showing Pearson's correlation of FC among each passage in the identical replicate under 40°C. A purple-to-orange color scale represents low to high Pearson's correlations. The lower panel shows a scatter plot comparing RPM before and after the 1st passage. (**G-H**) FC were quite reproducible between the replicates that originated from the identical pool, while they had some differences when the originating pools were different. Heatmaps showing Pearson's correlation among four replicates on the 10th passages at 30°C (**G**) and 40°C (**H**). "a-" and "b-" in the four replicates originated from Pool_a and Pool_b respectively. The comparisons **I-M** on the graphs **G** and **H** are shown as independent scatter plots in **I-M**.

I-M) Scatter plots comparing FC. The comparisons are described in G and H. "*r*", "*n*", and "*p*" mean correlation coefficients, sample number, and p-values, respectively. "$p \sim 0$" indicates that the *p*-value is smaller than the value that can be calculated.
