# Supplementary material for: Overexpression profiling reveals cellular requirements in the context of genetic backgrounds and environments
Source: PLoS Genet. 2023 Apr 28;19(4):e1010732. doi: 10.1371/journal.pgen.1010732 (PMC10171610; doi:10.1371/journal.pgen.1010732)
Supplement: S5 Fig — (PDF) [file pgen.1010732.s005.pdf]

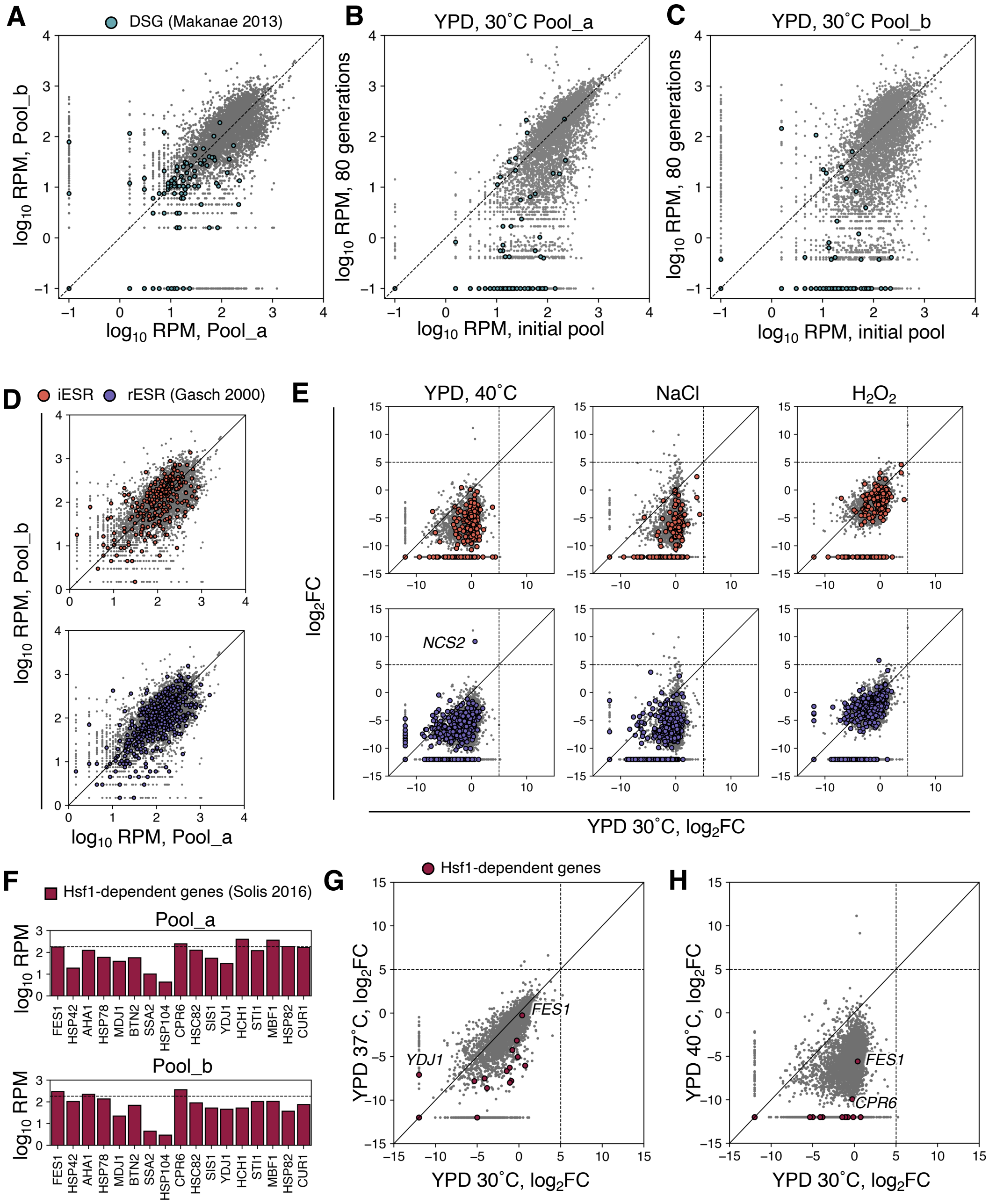

S5 Fig. Behaviors of dosage-sensitive genes and stress-induced genes in overexpression profiling.

(**A-C**) RPM of dosage-sensitive genes (DSG, green circles) [1] in the initial Pool_a and Pool_b (A), and after 80 generation cultivation in YPD at 30°C in Pool_a (B) and Pool_b (C). (**D**) Initial RPM of induced environmental stress response genes [2] (iESR, top, and orange circles) and reduced ESR (rESR, bottom, and purple circles) in Pool_a and Pool_b.

The grey circles represent genes other than ESR. (**E**) The scatter plots show comparisons of the fold change of iESR (top and orange circles) and rESR (bottom and purple circles) between 30°C and other three conditions: 40°C (left), 1 M NaCl (middle), and 2 mM H2O2 (right). The grey circles represent genes other than ESR. (**F**) Initial RPM of heat shock response genes (Hsf1-dependent genes [3]) in Pool_a (top) and Pool_b (bottom). (**G-H**) The scatter plots show comparisons of FC of Hsf1-dependent genes between 30°C and (**G**) 37°C or (**H**) 40°C. The red and grey circles represent Hsf1-dependent genes and other genes respectively. All circles are the mean FC of four replicates. The horizontal and vertical dashed lines indicate the threshold of GOFAs ($log_2FC \geq 5$). Genes with log_2 FC of nan (undetected) have -12 added for convenience.

Reference

1. Makanae K, Kintaka R, Makino T, Kitano H, Moriya H. Identification of dosage-sensitive genes in *Saccharomyces cerevisiae* using the genetic tug-of-war method. Genome Res. 2013;23: 300–311.

2. Gasch AP, Spellman PT, Kao CM, Carmel-Harel O, Eisen MB, Storz G, et al. Genomic expression programs in the response of yeast cells to environmental changes. Mol Biol Cell. 2000;11: 4241–4257.

3. Solís EJ, Pandey JP, Zheng X, Jin DX, Gupta PB, Airoldi EM, et al. Defining the Essential Function of Yeast Hsf1 Reveals a Compact Transcriptional Program for Maintaining Eukaryotic Proteostasis. Mol Cell. 2018;69: 534.
