# Supplementary material for: Overexpression profiling reveals cellular requirements in the context of genetic backgrounds and environments
Source: PLoS Genet. 2023 Apr 28;19(4):e1010732. doi: 10.1371/journal.pgen.1010732 (PMC10171610; doi:10.1371/journal.pgen.1010732)
Supplement: S6 Fig — (PDF) [file pgen.1010732.s006.pdf]

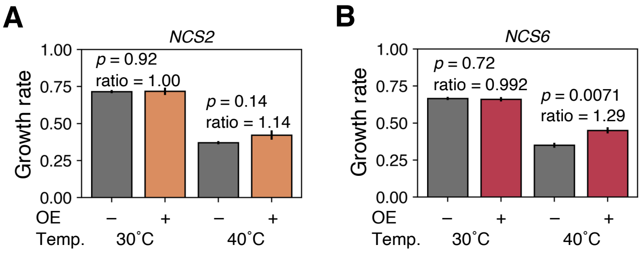

S6 Fig. Effect of overexpression of *NCS2* and *NCS6* at high temperature.

(**A-B**) Maximum growth rates of BY4741 cells overexpressing (OE) *NCS2* (**A**) and *NCS6*. (**B**) at 30°C and 40°C. The p-values are from two-tailed Welch's t-test (n = 3). Ratios were the average growth rate over the empty vector control. Error bars show standard deviation (SD) (n = 3).
