# Supplementary material for: Overexpression profiling reveals cellular requirements in the context of genetic backgrounds and environments
Source: PLoS Genet. 2023 Apr 28;19(4):e1010732. doi: 10.1371/journal.pgen.1010732 (PMC10171610; doi:10.1371/journal.pgen.1010732)
Supplement: S7 Fig — (PDF) [file pgen.1010732.s007.pdf]

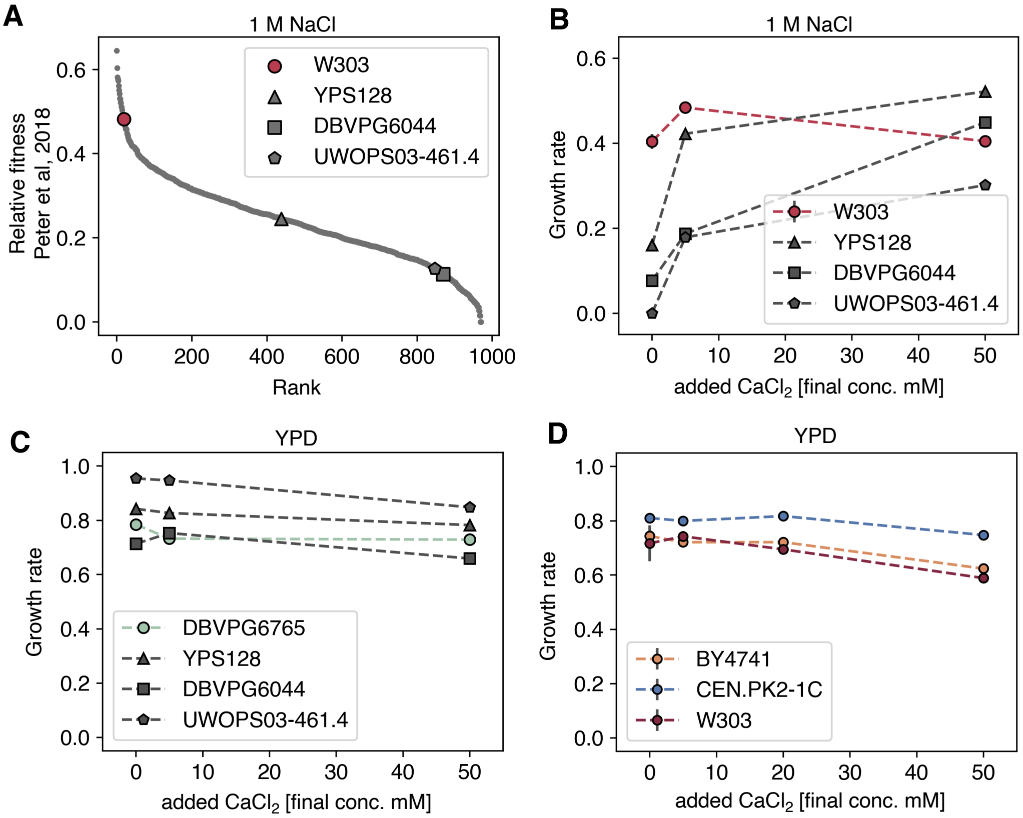

S7 Fig. The addition of Ca^{2+} increased the growth rates of various strains under salt stress but not without salt stress.

(**A**) Relative fitness of various strains; W303, YPD128, DBVPG6044, and UWOPS03-461.4 under 1M NaCl. The relative fitness data were from [1]. (**B**) Relationship between the addition of $CaCl_2$ and growth rates of various strains under 1 M NaCl. The growth rate of UWOPS03-461.4 without $CaCl_2$ addition (no growth) is set to 0 for convenience. Three biological replicates were measured for W303. (**C**) Relationship between the addition of $CaCl_2$ and growth rates of various strains under YPD. (**D**) Relationship between the addition of $CaCl_2$ and growth rates of various strains; BY4741, CEN.PK2-1C, and W303 under YPD. Error bars indicate standard deviation (SD) ($n = 3$). There are no significant differences between adding Ca or not (Welch's t-test and Bonferroni correction ($p \leq 0.05/3$)).

Reference

1. Peter J, De Chiara M, Friedrich A, Yue J-X, Pflieger D, Bergström A, et al. Genome evolution across 1,011 *Saccharomyces cerevisiae* isolates. Nature. 2018;556: 339–344.
