# Supplementary material for: Overexpression profiling reveals cellular requirements in the context of genetic backgrounds and environments
Source: PLoS Genet. 2023 Apr 28;19(4):e1010732. doi: 10.1371/journal.pgen.1010732 (PMC10171610; doi:10.1371/journal.pgen.1010732)
Supplement: S8 Fig — (PDF) [file pgen.1010732.s008.pdf]

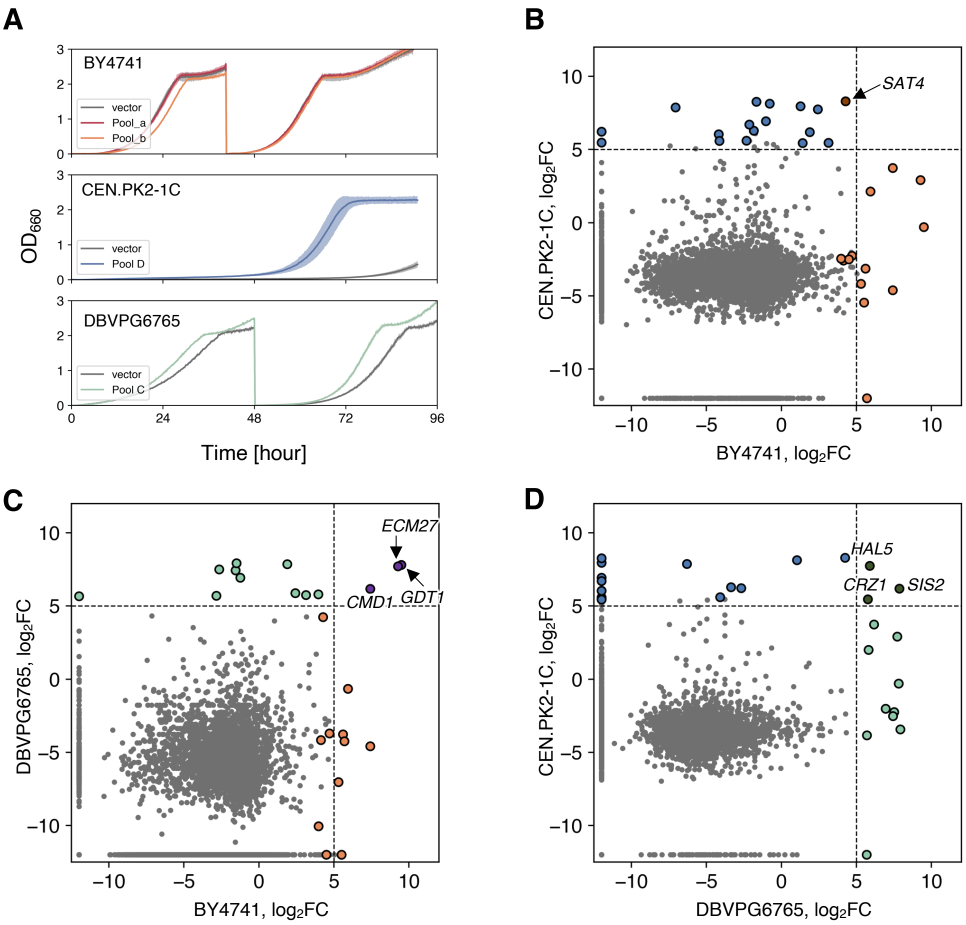

S8 Fig. Supplement to overexpression profiling of CEN.PK and DBVPG6765.

(**A**) Overexpression libraries of CEN.PK2-1C (middle, blue) and DBVPG6765 (bottom, light green) grew faster than the vector controls and quickly adapted to the salt stress. The solid lines and the filled areas indicate the average of OD_{660} and the standard deviation (n = 3) respectively. The grey lines represent the empty vector controls. The top panel shows the growth curves of BY4741 library under salt stress. The red and orange correspond to replicates derived from Pool_a and Pool_b (each n = 2). (**B-D**) Scatter plots show comparisons of the average FC between BY4741 and CEN.PK2-1C (**B**), BY4741 and DBVPG6765 (**C**), and DBVPG6765 and CEN.PK2-1C (**D**), under 1M NaCl. The orange, blue, and light green circles indicate GOFAs in BY4741, CEN.PK2-1C, and DBVPG6765 respectively. The brown, purple, and green circles represent multiple hit genes in BY4741 and CEN.PK2-1C, BY4741 and DBVPG6765, and CEN.PK2-1C and DBVPG6765 respectively. Genes with a log_2FC of nan (undetected) have -12 added for

convenience. The horizontal and vertical dashed lines indicate the threshold of GOFAs ($log_2FC \geq 5$).
