# Supplementary material for: Overexpression profiling reveals cellular requirements in the context of genetic backgrounds and environments
Source: PLoS Genet. 2023 Apr 28;19(4):e1010732. doi: 10.1371/journal.pgen.1010732 (PMC10171610; doi:10.1371/journal.pgen.1010732)
Supplement: S9 Fig — (PDF) [file pgen.1010732.s009.pdf]

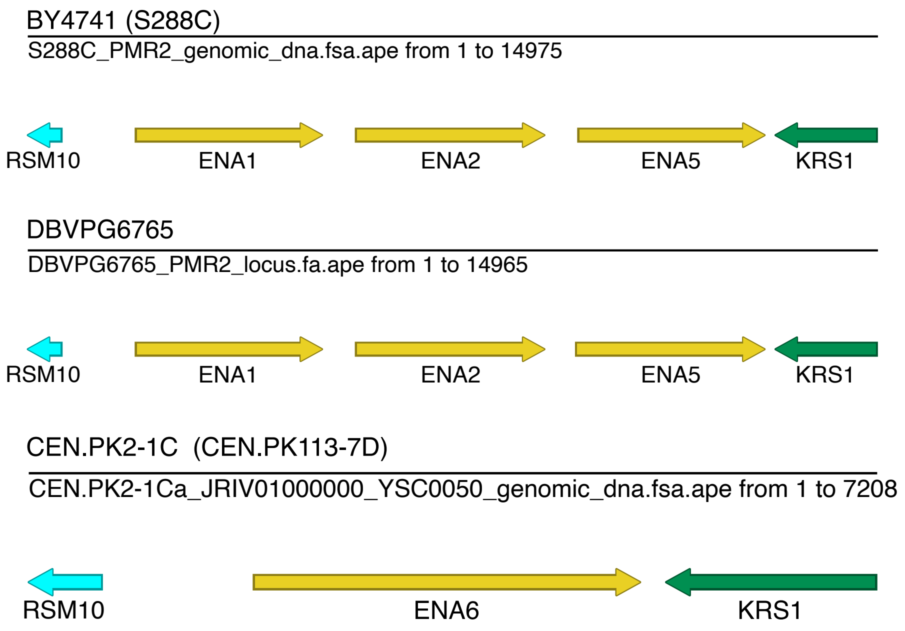

S9 Fig. Schematic diagram of the *PMR2* locus in BY4741, DBVPG6765, and CEN.PK.
