# Supplementary material for: Overexpression profiling reveals cellular requirements in the context of genetic backgrounds and environments
Source: PLoS Genet. 2023 Apr 28;19(4):e1010732. doi: 10.1371/journal.pgen.1010732 (PMC10171610; doi:10.1371/journal.pgen.1010732)
Supplement: S10 Fig — (PDF) [file pgen.1010732.s010.pdf]

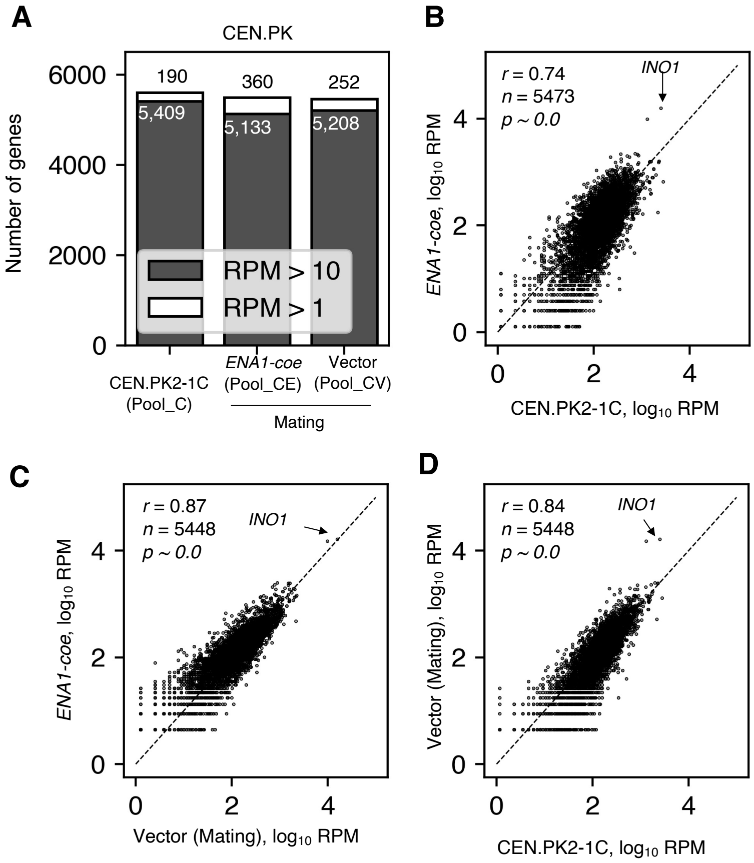

S10 Fig. The quality check of the CEN.PK2-*ENA1* co-overexpression library.

(**A**) CEN.PK2-*ENA1* co-overexpression (-coe) libraries covered over 5,000 genes in CEN.PK. The filled and open bars indicate RPM ≥ 10 and RPM ≥ 1 respectively. (**B-C**) Scatter plots show comparisons of initial RPM between (**B**) CEN.PK2-1C and CEN.PK2-*ENA1*-coe, (**C**) CEN.PK2-*ENA1*-coe and vector, and (**D**) CEN.PK2-1C and vector. As an addendum, we found that *INO1* was enriched in these libraries during construction and might compensate for the lack of Inositol in the SC medium used in the selection [1].

Reference

1. Hanscho M, Ruckerbauer DE, Chauhan N, Hofbauer HF, Krahulec S, Nidetzky B, et al. Nutritional requirements of the BY series of *Saccharomyces cerevisiae* strains for optimum growth. FEMS Yeast Res. 2012;12: 796–808.
