# Supplementary material for: Overexpression profiling reveals cellular requirements in the context of genetic backgrounds and environments
Source: PLoS Genet. 2023 Apr 28;19(4):e1010732. doi: 10.1371/journal.pgen.1010732 (PMC10171610; doi:10.1371/journal.pgen.1010732)
Supplement: S11 Fig — (PDF) [file pgen.1010732.s011.pdf]

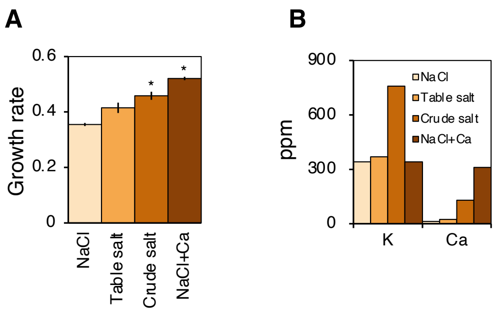

S11 Fig. Effect of different salt sources on the growth of yeast.

(**A**) Growth rate of BY4741 under three different salt sources: NaCl as an experimental reagent, table salt, and crude salt. NaCl+Ca means NaCl medium adding 5 mM $CaCl_2$. Asterisks indicate significant differences compared to NaCl (Welch's t-test and Bonferroni correction ($p \leq 0.05/3$)). (**B**) K^+ and Ca^{2+} concentrations in the medium used in **F**. Na^+ concentrations in all mediums were adjusted to 23.0 g/l. Error bars indicate SD ($n = 3$).
