# Supplementary material for: Overexpression profiling reveals cellular requirements in the context of genetic backgrounds and environments
Source: PLoS Genet. 2023 Apr 28;19(4):e1010732. doi: 10.1371/journal.pgen.1010732 (PMC10171610; doi:10.1371/journal.pgen.1010732)
Supplement: S12 Fig — (PDF) [file pgen.1010732.s012.pdf]

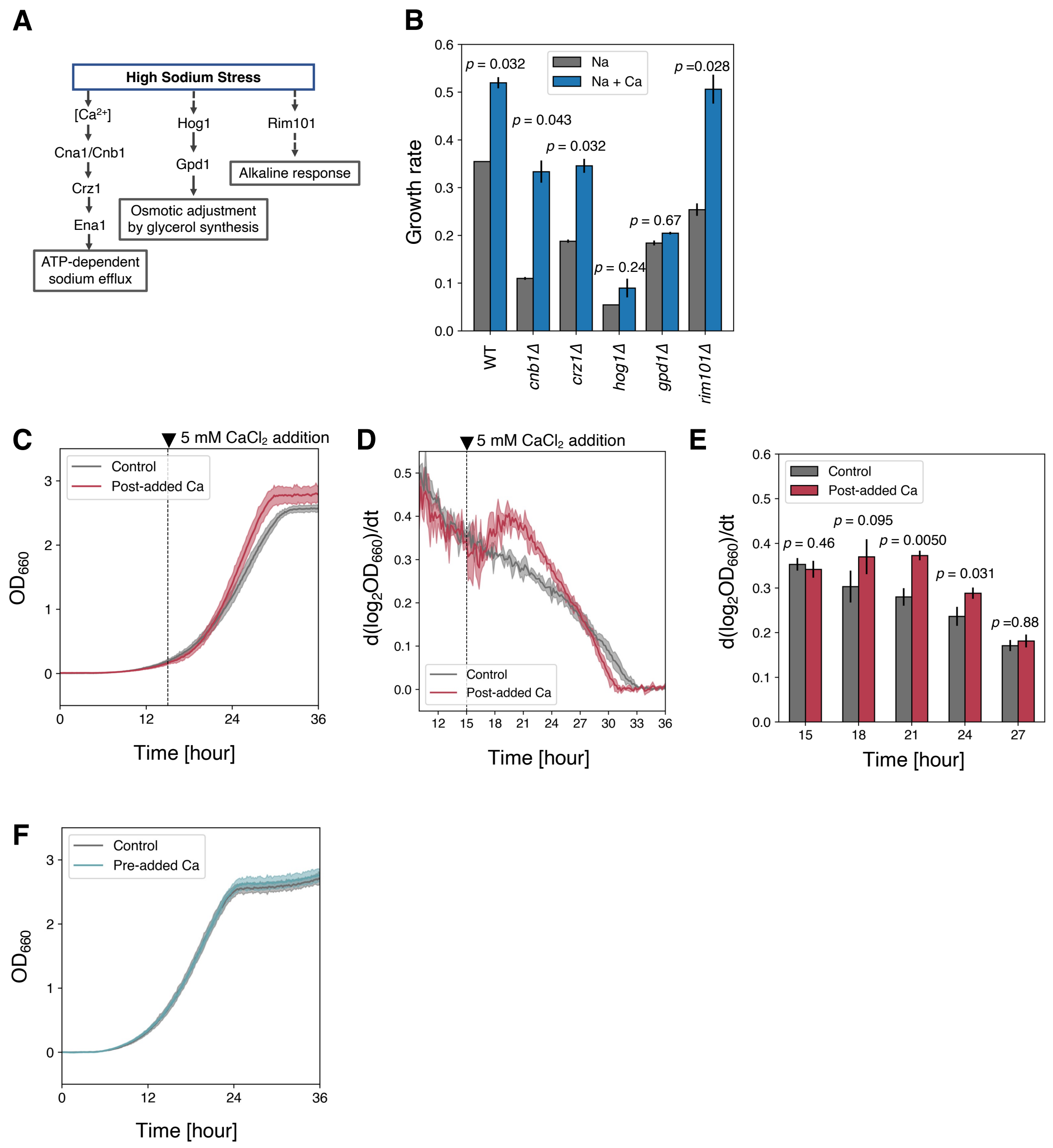

S12 Fig. The effects of calcium addition alone cannot be explained by short-term stress response enhancement.

(**A**) A scheme of major responses to salt stress. (**B**) Growth rates of knockouts related to major responses to salt stress. The grey and blue bars indicate growth rates under 1 M NaCl with/without 5 mM CaCl2 respectively. Error bars indicate SD ($n = 2$). The p-values are from two-tailed Welch's t-test ($n = 2$). (**C-E**) Growth of BY4741 under salt stress with $CaCl_2$ added after salt stress exposure. The growth curves of BY4741 under 1 M NaCl and adding 5 mM $CaCl_2$ after the salt stress exposure 15 hours later (**C**). Instantaneous growth rates per hour (**D**) and their comparison every 3 hours (**E**). The vertical dashed

line indicates the timing of $CaCl_2$ addition. The filled areas and error bars indicate standard deviation (SD) (n = 3). The p-values are from Welch's t-test. (**F**) The growth curves of BY4741 under 1 M NaCl and adding 5 mM CaCl2 at pre-cultivation but not during the salt stress. The filled areas indicate SD (n = 3).
