# Supplementary material for: Overexpression profiling reveals cellular requirements in the context of genetic backgrounds and environments
Source: PLoS Genet. 2023 Apr 28;19(4):e1010732. doi: 10.1371/journal.pgen.1010732 (PMC10171610; doi:10.1371/journal.pgen.1010732)
Supplement: S13 Fig — (PDF) [file pgen.1010732.s013.pdf]

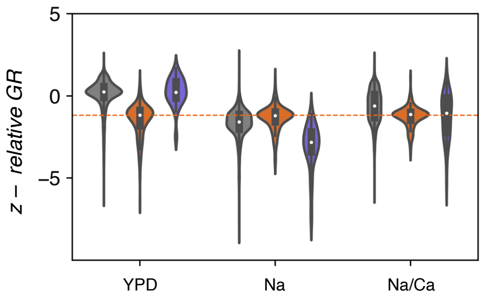

S13 Fig. Relative fitness distribution when the growth rates in each condition were subtracted from Fig 5F.
