# Supplementary material for: Overexpression profiling reveals cellular requirements in the context of genetic backgrounds and environments
Source: PLoS Genet. 2023 Apr 28;19(4):e1010732. doi: 10.1371/journal.pgen.1010732 (PMC10171610; doi:10.1371/journal.pgen.1010732)
Supplement: S14 Fig — (PDF) [file pgen.1010732.s014.pdf]

A

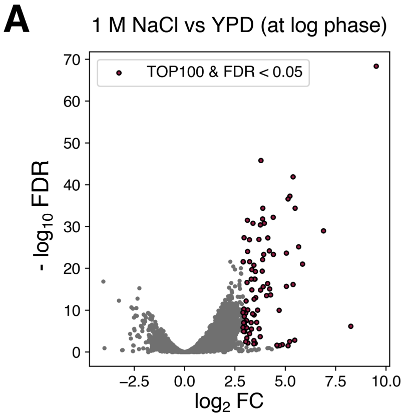

B

Biological process	GOID	Match
response to oxidative stress	GO:0006979	9/88
arginine biosynthetic process	GO:0006526	4/88
cellular response to chemical stress	GO:0062197	9/88
cellular response to oxidative stress	GO: 0034599	8/11
**Cellular component**	**GOID**	**Match**
-	-	-
**Molecular function**	**GOID**	**Match**
-	-	-

C

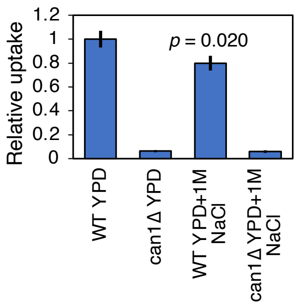

D

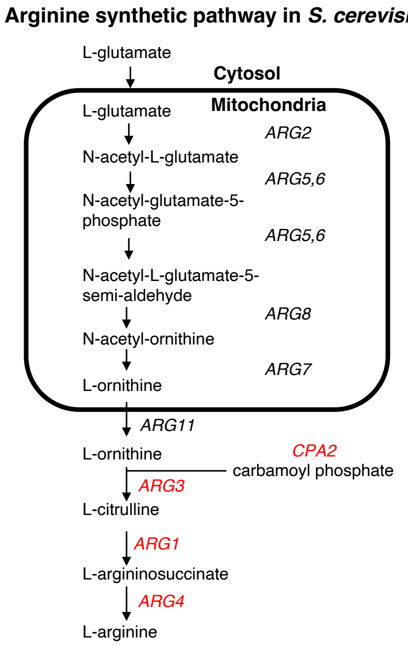

E

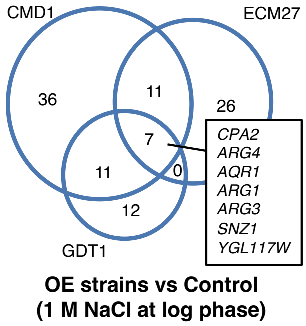

S14 Fig. Transcriptome analysis of cells under salt stress obtained by RNAseq analysis.

(**A**) Transcriptome changes in 1 M NaCl and YPD at log growth phase. ORFs with the top 100 most enormous fold changes and significant changes (FDR ≤ 0.05) compared with YPD are highlighted. (**B**) Gene ontology terms enriched in highlighted ORFs are shown in the left table. (**C**) Arginine intake assay under YPD and 1 M NaCl. The *p*-values are from Welch's t-test ($n = 3$). The error bars indicate SD. (**D**) Scheme showing arginine synthesis pathway in *S. cerevisiae*. Red-colored genes were upregulated under 1 M NaCl and downregulated in GOFA's overexpression stains. (**E**) Venn diagram showing

downregulated genes (FDR ≤ 0.05) in GOFA's overexpression stains. These data are summarized in S8 Table.
