# Supplementary material for: Overexpression profiling reveals cellular requirements in the context of genetic backgrounds and environments
Source: PLoS Genet. 2023 Apr 28;19(4):e1010732. doi: 10.1371/journal.pgen.1010732 (PMC10171610; doi:10.1371/journal.pgen.1010732)
Supplement: S15 Fig — (PDF) [file pgen.1010732.s015.pdf]

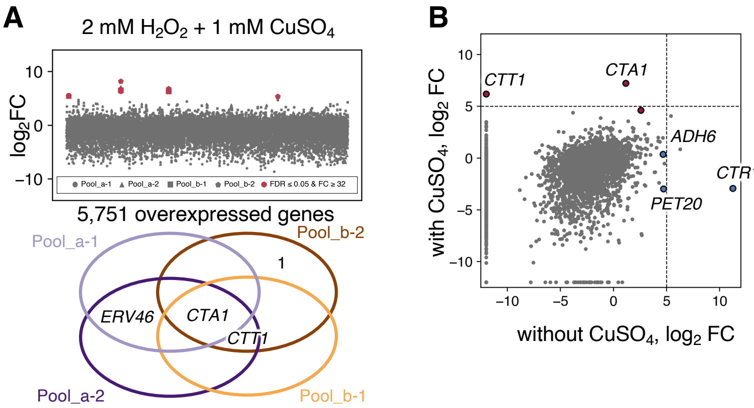

S15 Fig. GOFAs enriched under oxidative stress propose Cu^{2+} limitation in the culture medium.

(**A**) Fold change of plasmid occupancy after the 80 generations-cultivation of BY4741 overexpression library under 2 mM H_2O_2 with 1 mM $CuSO_4$. (upper). Hit genes in each replicate under well-studied stresses (lower, FDR ≤ 0.05 and $log_2FC \geq 5$). The log2FC is plotted along the y-axis as a function of the 5,751 overexpressed genes ordered by ORF names. Hits are summarized in S7 Table. (**B**) Compare the fold changes of plasmid occupancies with and without $CuSO_4$ addition. The colored circles indicate multiple hit genes with (red) and without $CuSO_4$ addition (blue). The dashed lines mean the threshold of GOFAs as $log_2FC \geq 5$. No values were replaced by -12.
