# Supplementary figures and images for: Overexpression profiling reveals cellular requirements in the context of genetic backgrounds and environments
Source: PLoS Genet. 2023 Apr 28;19(4):e1010732. doi: 10.1371/journal.pgen.1010732 (PMC10171610; doi:10.1371/journal.pgen.1010732)

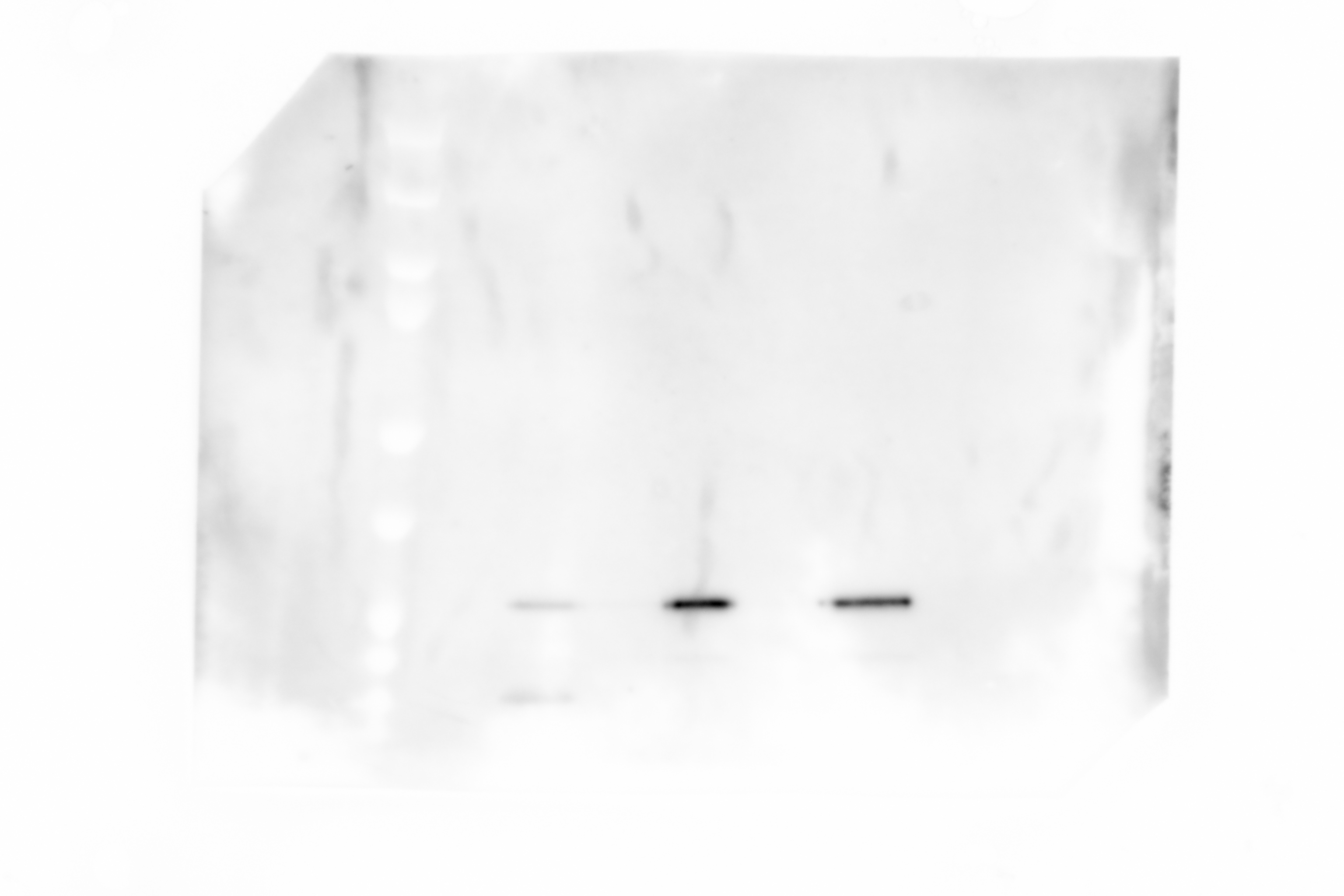

Supplement: S1 Data — (ZIP) [file pgen.1010732.s025.zip › S1_Data/Fig_5/Fig.5A/Fig.5A_Rep2]

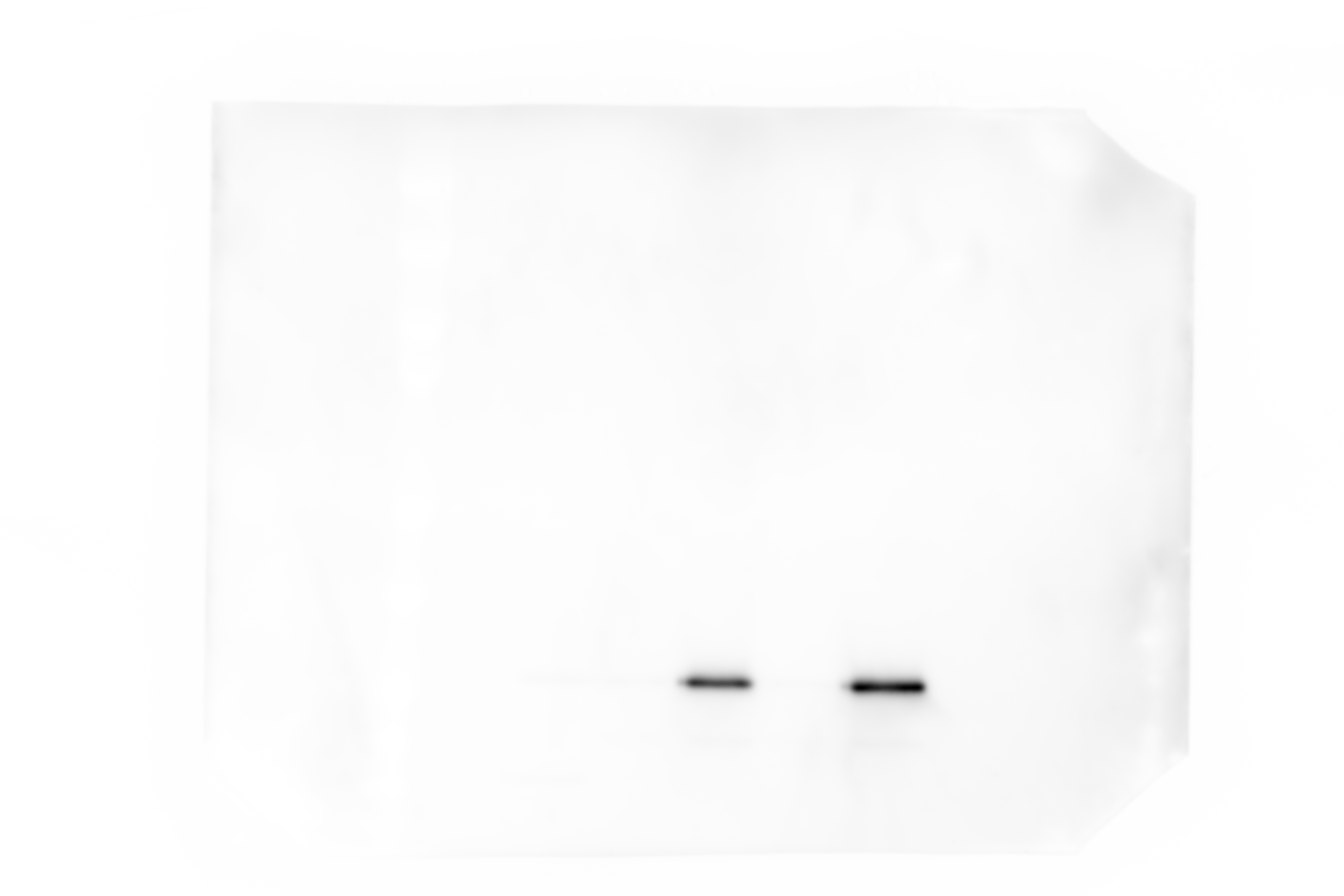

Supplement: S1 Data — (ZIP) [file pgen.1010732.s025.zip › S1_Data/Fig_5/Fig.5A/Fig.5A_Rep3]

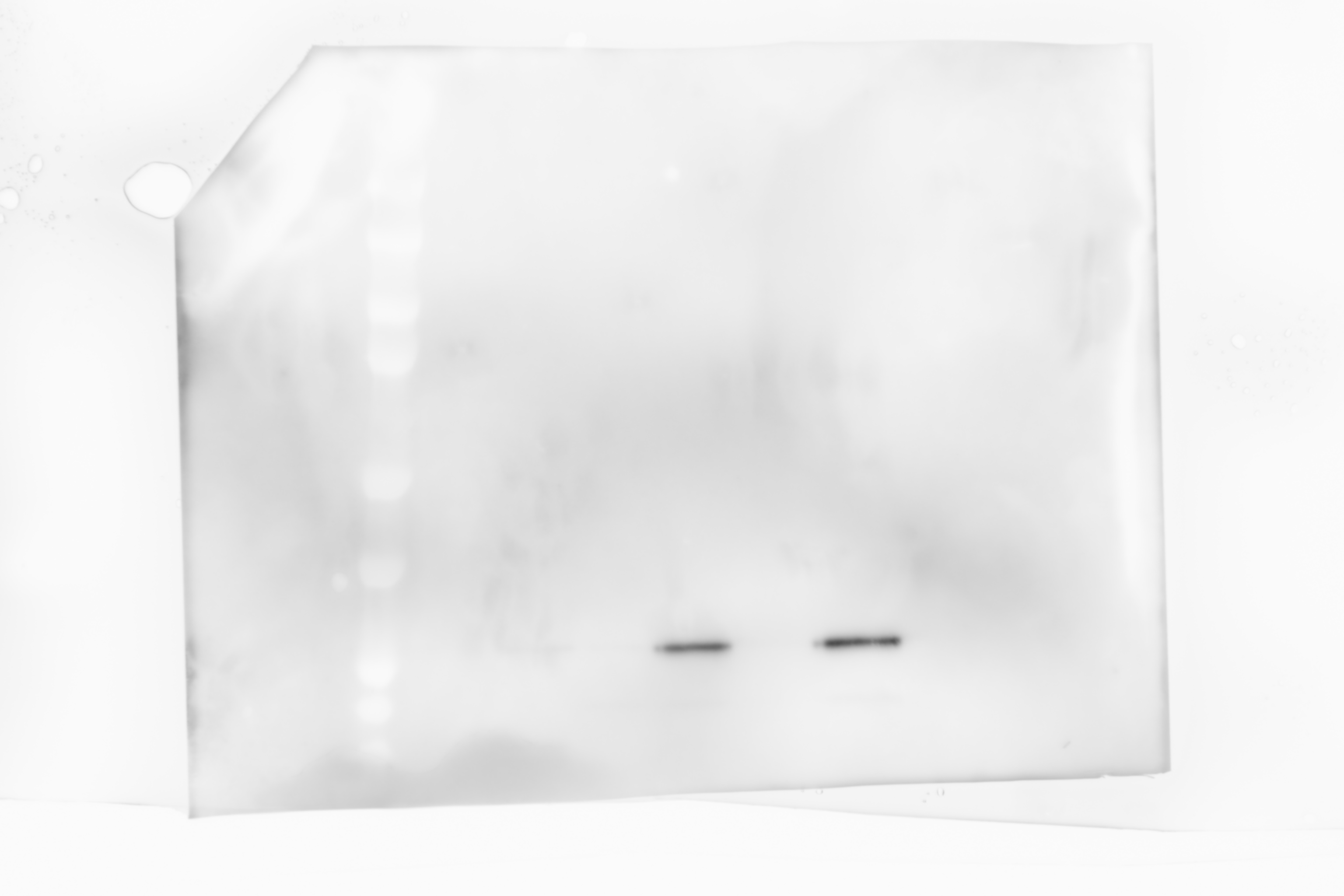

Supplement: S1 Data — (ZIP) [file pgen.1010732.s025.zip › S1_Data/Fig_5/Fig.5A/Fig.5A_Rep1]
