# Supplementary material for: Overexpression profiling reveals cellular requirements in the context of genetic backgrounds and environments
Source: PLoS Genet. 2023 Apr 28;19(4):e1010732. doi: 10.1371/journal.pgen.1010732 (PMC10171610; doi:10.1371/journal.pgen.1010732)
Supplement: Attachment — Submitted filename: Response_to_reviewer_20230130.docx [file pgen.1010732.s026.docx]

We greatly appreciate the reviewers' thorough and constructive comments. We have thoroughly revised our manuscript based on their feedback, particularly regarding the statistical explanation of GOFA identification and the Introduction. We believe our manuscript has greatly improved from its original version. Below is a point-by-point response to the reviewer's comments.

**Response to Reviewer #1**

*Many key experiments in the manuscript lack statistical analysis. In particular, the hits from the overexpression profiling were determined using an enrichment threshold following selection, but the authors generally did not calculate a false discovery rate associated to the threshold (the one exception being fig. 5D). The authors performed four replicate experiments, but it does not appear that they leveraged the replicates to determine statistical significance.*

We had also wondered if statistical analysis could be applied. However, we realized that this experimental system, as shown in Figure 1C, resulted in a rapid transition from a diverse plasmid population to one dominated by a small number of highly enriched plasmids and a large number of lost plasmids. This makes statistical comparisons between the initial pool (or the pool under experimental conditions where no enrichment occurred) and the adapted one almost meaningless. On the other hand, we concur that there is a need to objectively demonstrate the situation through statistical analysis. We thus considered using the chi-square test and its Benjamini-Hochberg correction for false discovery rate calculation to express the significance of changes in each gene in the competition experiments mentioned above as a statistical number.

We first modeled the task of randomly sampling specific plasmids from the plasmid pool and determining their frequency of occurrence as a binomial distribution. Due to the large number of trials (over 10^5), we approximated it using a chi-square test. We then applied a multiple test correction using the Benjamini-Hochberg method to calculate the false discovery rate (FDR). As expected, most genes showed significant FDRs (≤ 0.05) in the sample after the competition experiment. Then, we considered those genes with FDRs ≤ 0.05 and log_2_FC ≥ 5 as "hits" - potential candidates for GOFA. We also used a Venn diagram to show replicates, and those obtained with multiple replicates were considered confident GOFAs. We also created a Venn diagram to visualize the replicates, and those identified in multiple replicates were considered as confident GOFAs.

A detailed explanation of this statistical analysis was added to the Materials and Methods section. We replaced the original circular charts with dot plots and Venn diagrams (Figures 1D-H, F3E-G, 4B, 6B, S15A) to present the results.

*The authors quantify the abundances of their overexpression constructs following selection as “degree of enrichment,” which I interpreted as a measure of fold-change following selection, so the threshold used by the authors, 10,000, seemed like quite an impressive bar to clear. But “degree of enrichment” is not actually defined in the Results section (I recommend doing so); in the Methods section it is described on line 618 as “the number of plasmids per million plasmids after [selection], assuming that the initial pool was completely uniform.” I would not call that a “degree of enrichment,” since it does not reflect fold-change, that is, enrichment. Probably a clearer description would be “normalized reads per million.” But why not use a simple fold-change measure?*

We initially did not want to use fold change (FC) because FC = 1 does not mean “neutral” because most neutral genes should be dropped-out during competition (thus they should show FC < 1). However, as the objective of our study is to focus on enriched genes (i.e. genes with high fold increase), we decided to use fold change as a measure as suggested by the reviewer.

We rewrote the text and figures to replace the previously used measure (DoE) with fold change.

*The manuscript seems to have an error when defining the “degree of enrichment” discussed above, which further confuses the matter. An equation is given in the Methods (line 616) for calculating this value, but a different equation is given in Extended Data Figure 3C; the two equations differ by a factor of 10^6. I suspect the equation in the Methods section is incorrect and the one in the Extended Data Figure is correct. I also recommend using consistent nomenclature; “RPM” can be used in both places, instead of “p,” which is only used in the Methods.*

We apologize for any errors made. As stated above, we have decided to use fold change as the measure and deleted the pointed descriptions. Additionally, we have thoroughly reviewed and corrected any inaccuracies in our descriptions.

*The authors tested the overexpression library in additional strains. The Methods section describes the construction of this library as follows: “The mixed PCR products of S288C’s ORF and the two plasmid fragments from pTOW40836 were introduced into the target strain,” on line 685. I do not see any explanation of how this PCR was generated – what was the template? The primers? Was it a single PCR from some sort of DNA library, or separate PCRs that were pooled? Nor is there information on what the two plasmid fragments are. The Materials and Methods section needs to be detailed enough that a skilled independent investigator would be able to repeat the experiments with just the information provided. That is certainly not the case for this experiment, and I recommend the authors reconsider the entire Methods section with that standard in mind.*

The production of PCR products was previously reported in a study by Makanae et al. (2013) and thus was omitted in our paper. We apologize for any lack of detail in the description. We have now added the details of the production to the Material and Methods section. We have also thoroughly checked the description in the Material and Methods section to ensure that there are no other missing information and added any necessary details.

*The authors sequenced their control strains’ genomes following growth in salt stress, and mutations were identified in PMA1 and PMR1. (Section starting Line 652.) Were single colonies picked, or was the entire pool sequenced? Were these the only mutations identified?*

We performed whole-genome sequencing on pooled samples of the An and Ev strains and identified a total of six mutations (two on lineage 1 and four on lineage 2, **Table S3**). Four of the six mutations were identified in the coding regions of *PMA1*, *PMR1*, and *TRZ1*. One of these mutations, located in *TRZ1*, was also present in the ancestor. The other two mutations were found in intergenic DNA. A mutation in lineage 1 caused a P393Q substitution in Pma1, and two mutations in lineage 2 resulted in G428D and G824E changes in Pmr1 (**Figure 2J**).

We have included this description in the Results section.

*The authors provide p-values for luminescence intensity comparisons in figure 2D, but I could not tell what quantification of luminescence intensity was used in the statistical test, as the figures show values across a time window for each strain. Was the maximum value used? Or was it the area under the curve? Something else?*

We utilized the maximum values of luminescence for statistical testing. We included the statement, "Each maximum values of luminescence intensity was used for Welch's t-test." to the legend of Figure 2D.

*The authors state that there is “high energy demand under salt stress” (line 370). If this statement is based on work in the literature, a citation is needed. If the statement derives from results in this manuscript, I did not understand which results showed the statement to be true, so the basis for the statement should be more clearly explained.*

We cited the report by Olz *et al* (2013), which reported energy demands 28-51% increased under high salt stress over control.

*In Extended Data Figure 1, I think the letters printed over the squares in panels D and E are incorrect; I think the letters in panel D should be F, G, and H, and the letters in panel E should be I and J. The figure legend should explain what these letters indicate.*

We appreciate the reviewer’s detailed checking. We have corrected these errors.

*It would be helpful to see how the enrichment values correlate between replicate experiments. In Extended Data Figure 1, in addition to showing the correlation of read counts between replicates, the authors should show the correlation between the calculated degree of enrichment.*

We included the correlation between the enrichment values (fold change) between replicates in Figure S4F-M.

*In Extended Data Figure 4, the authors follow the behavior of “dosage-sensitive genes.” If they are using designations from a different paper, the authors should provide a citation in the figure legend. It would also be helpful to provide a brief explanation of the experiment that was used to define genes as dosage sensitive.*

We have added a reference and brief explanation of dosage-sensitive genes in the main text as follows; “As expected, the dose-sensitive genes identified in our previous studies, those for which multiple copies have a negative impact on proliferation (Makanae *et al.*, 2013), were omitted (Figure S5A-C).”

*Extended figure 5F says that “asterisks indicate significant differences compared to NaCl,” but I do not see any asterisks? There are also no error bars.*

We are sorry that Ex Figure.5F showing growth rates and Ex Figure.5G showing mineral concentrations were incorrectly interchanged. We have corrected the error (they are now Figure S11A and B).

*The circle plots (eg. Fig. 1D) are not very informative. The chromosomal coordinates aren’t useful information. It is not possible to tell what the median value is, etc. It is not clear to me that the circularity improves visualization over a linear layout. A scatterplot of values seems like it would convey more information.*

We prefer using circular plots because they provide an intuitive representation of how certain genes are enriched (and others are lost) after competitive culture. Additionally, the chromosomal locations of genes that are enriched in multiple conditions can be easily recognized. A typical example is shown below. In YPD at 40 ˚C, *NCS2* and *NCS6* are strongly enriched, and their enrichments are also present in YPD at 37 ˚C, but to a lesser degree. With circular plots, this information is easily and intuitively recognizable, whereas it is not as clear with dot plots as shown below.

However, we acknowledge that dot plots are better suited for displaying replicated data and fold change. Therefore, we have decided to abandon the use of circular plots and adopt dot plots as suggested by the reviewer.

*I think the authors invent too many acronyms over the course of the manuscript (ADOPT, DoE, GOFA, ADOPT 2.0, ADOPT 2.1). This is especially confusing when the name of the acronym isn’t very informative. For instance, “autonomous dosage-optimization using plasmids with two-micron origin” is not at all a clear description of the method, and the word “ADOPT” has no relation to what it is describing. Please consider switching to standard informative terms like “overexpression profiling” for “ADOPT” and “hits” for “GOFAs.”*

We removed most acronyms, except for GOFA, as it contains the main concept we propose in this manuscript and is used frequently throughout. While "ADOPT" (autonomous dosage-optimization using plasmids with two-micron origin) describes the mechanism of our screening system, we did not explain it in our manuscript, we therefore chose not to use it. Instead, we decided to use "overexpression profiling" as suggested by the reviewer, while it is a more general term that does not specify a specific experimental system.

*There are various grammatical errors or word choice issues. A few points I noticed:…*

We appreciate the reviewer's attention to detail and apologize for any errors in our previous submission. We have thoroughly reviewed our English and made the necessary revisions.

**Response to Reviewer #2**

*1. I think the authors should include a better background overview of yeast traits and how these polygenic are influenced by different genes. There are several studies in the gene deletion collection and QTL mapping studies providing evidence of the genetics underlying these traits. That being said, what’s missing? What’s the motivation for your study? I believe this is missing in your INTRODUCTION section and is dispersed across the results section.*

Introduction was largely rewritten to include that information.

*2. Throughout the results section different comparisons are made, but the statistics are generally missing. Either correlations, or comparisons between pools do not contain the statistics in the text or figures. Please include them every time to make sure that differences are significant (i.e L221, L227…).*

We thoroughly reviewed the results section and incorporated appropriate statistics. For instance, we performed statistical tests to compare the growth rates between strains (corresponding to L221 and L227 in previous manuscripts, as shown in Figure 3A) and examined the correlations in the frequency of plasmid appearance (new Figure S3 and Figure S4F-H).

*3. Based on their G x E data, the authors could estimate for each overexpressed gene from their assay the estimated effect on each case using a linear model: lm (OE~environment x strain). In this way, for each gene they could actually assess G x E based on their title.*

Estimating the effect of G x E would be interesting, but I am unsure of how to calculate it using our current data set. If we had access to more extensive and large-scale sequencing, it may have been possible to calculate the relative adaptation in (OE ~ environment x strain) as demonstrated in Levy *et al'*s study in *Nature*, 2015, by analyzing the frequency of plasmid appearance in the time series. We acknowledge this as a potential future issue and have not included it in our current manuscript.

*L49. Protect what cell function?*

*L54. Please provide examples.*

*L62. Please expand on polygenic traits and how genes and genetic interactions underlie different phenotypes (such as heat or NaCl)*

We completely rewrote the Introduction section and incorporated responses to the comments.

*L117-120. Examples of genes are given, why those ones? Function of DoE?*

We clarified the reasoning behind the inclusion of these genes in the revised manuscript. These genes were identified as hits in all four replicates and met the new thresholds (FDR ≤ 0.05, log2FC ≥ 5) established during the revision process.

*L132. Remove ‘first’ if no other ‘second’ or ‘next’ is mentioned in this section.*

The 'first' is linked to the 'next' in the following section.

*L156. Authors mentioned that Ca+ increases the growth rate, but when observing figure2E I see something different, growth rate goes down. Am I missing something?*

We apologize for the confusion in our original presentation. We meant to describe “The addition of Ca2+ up to 20 mM in a 1M NaCl medium was found to enhance the growth rate of cells under salt stress, as shown in Figure 2E. However, further increases above 40 mM led to a decrease in growth”. We revised the description as above.

*L164. Normally these mutations are expressed as ‘P393Q’, and so on.*

We revised the expressions as pointed.

*L194-197. Please specify backgrounds and include this information in the introduction section*

We included this information in the Introduction section.

*L238. Please indicate that a smaller number of generations was considered in this case.*

We included the description as follows in the corresponding place. “Due to their faster growth compared to the control, we shortened the duration of the competitive culture for CEN.PK and DBVPG6765 in comparison to BY4741, terminating it after 8 and 16 generations, respectively.”

*L240. Authors should consider the greater overlap between CEN.PK and DBVPG6765*

Because we re-analyzed hits in response to the comment of Reviewer #1, we have revised Figure 3H, and the corresponding description as follows. “Interestingly, most of the identified genes were specific to a single strain and only a few genes appeared in more than two strains.”

*L243. Sometime is better to use the term ‘genetic background’ than ‘strain’.*

We have changed “strain” to “genetic background” in the corresponding places.

*L339. Authors should make sure that these part of the story is focused on mutants, and the lack of certain genes.*

We revised the first sentence as “We thus focused on the knockout strains of mitochondrial (Mito) genes”.

*L365. Please describe how did you ‘observed’ mitochondria.*

We had described it in the legend of Figure 5K as “The green color shows the mitochondria inner membrane observed with Tim50-GFP. The red color indicates the mitochondrial ROS level stained by MitoTracker™ Red CM-H2Xros.”

*L404. Rephrase ‘only about 20%’. Was significantly reduced only 20%?*

We rephrased to “Additionally, we found that arginine uptake was significantly reduced by 20% under salt stress conditions (Figure S14C).”

*L526. Remove RNA-Seq*

We removed it.

*L530. Not sure these should be in methods.*

We decided to keep them.

**Response to Reviewer #3**

*However, regarding the form of the manuscript, I find the introduction really weak, and the authors should make an effort to present a more complete state-of-the-art on their topic. Both aspects of adaptive processes (through gene overexpression, but not only) in yeast and strategies developed to test (genome-wide) gene over-expression should at least be developed.*

Introduction was largely rewritten to include that information.

*As mentioned by the authors, in some cases, control strains evolved and became more adapted than the “ADOPTed” ones (fig2G). This also suggests that, within the ADOPT libraries, primary mutations affecting a gene unrelated to the overexpressed one could lead to plasmid enrichment in the final culture. This factor is only poorly controlled in their experiments. The availability of replicates should be leveraged to counterpart this type of bias.*

We appreciate this suggestion.

We included explanations that we used multiple replicates to avoid any effect of primary mutations as follow.

“We believe that repeating experiments is crucial in distinguishing between adaptive mutant cells that emerged randomly during competitive culture and adaptive cells that overexpress specific genes that were present in the pool from the start. Specifically, if the same overexpression strain is enriched after multiple independent cultures, it strongly suggests that the overexpression strain was adaptive rather than resulting from mutation.”

We also emphasized that we used freshly prepared plasmid transformants for our evaluation experiments in the text as follows.

“It is worth noting that, to verify the function of GOFAs, we employed the use of freshly prepared plasmid transformants that had not been exposed to stress conditions in all experiments. This approach was taken to confirm that any observed phenotypes were a direct result of the overexpression of specific genes rather than mutations that may have occurred during adaptation to stress or storage.”

Additionally, we have included Venn diagrams to show the reproducibility of the isolation of hits, in response to the comment of Reviewer #1.

*There is no mention of the potential false positive GOFAs that might be highlighted, and why they would appear. This should be discussed at some point.*

We included this discussion in the Discussion section as follows. “We noticed that there were some technical limitations or biases when trying to isolate true GOFAs using our approach. 1) The competitive culture potentially accumulates genomic mutations that are adaptive to the stress, as observed in Figure 2G. 2) Isolation of genes was influenced by the initial pools (Figure S3) because of the potential presence of preexisting genomic mutations or variations in the starting occupancy of cells with each plasmid. 3) Plasmid sequencing has a bias towards certain insert lengths (Figure S4). To minimize these biases and obtain confident GOFAs, we employed several strategies."

*l.107, the authors stated that “Enriched genes were highly reproducible across replicates”, while the plots they refers to do mostly not show that trend, in particular between the pools of different origins. This is even more visible in fig 2A.*

We rewrote this section in detail after reanalyzing our data in response to Reviewer #1's comments, specifically as follows. “Overall, plasmid reads from replicates gave satisfactory reproducibility (Pearson's *r* > 0.4), but there was a higher correlation between the duplicates from the same pool than between duplicates from different pools (Figure S3).”

*for most of the analyses, the authors consider as DoE the mean value of the 4 replicates. With this method, the reproducibility is not taken into account per se, and some bias related to the aforementioned phenomena may persist. The author should propose a way to seriously take the replicates into account in their strategy, or at least justify their choice.*

The same concern was mentioned by Reviewer #1 and described in the first response of its response as well. We first modeled the task of randomly sampling specific plasmids from the plasmid pool and determining their frequency of occurrence as a binomial distribution. Due to the large number of trials (over 10^5), we approximated it using a chi-square test. We then applied a multiple test correction using the Benjamini-Hochberg method to calculate the false discovery rate (FDR). As expected, most genes showed significant FDRs (≤ 0.05) in the sample after the competition experiment. Then, we considered those genes with FDRs ≤ 0.05 and log_2_FC ≥ 5 as "hits" - potential candidates for GOFA. We also used a Venn diagram to show replicates, and those obtained with multiple replicates were considered confident GOFAs. We also created a Venn diagram to visualize the replicates, and those identified in multiple replicates were considered as confident GOFAs.

A detailed explanation of this statistical analysis was added to the Materials and Methods section. We replaced the original circular charts with dot plots and Venn diagrams (Figures 1D-H, F3E-G, 4B, 6B, S15A) to present the results.

*l.51: "accidental increases": accidental is maybe not an appropriate word here. Please reformulate.*

We thoroughly rewrote Introduction and removed this expression.

*l.129: why “unique”?*

We revised this expression as follows.

“These results suggest that the identified GOFAs are a distinct set of genes, which are not induced under stress but whose overexpression is adaptive.”

*l.133: Overexpression of NCS2 and NCS6 (-oe) independently resulted in increased heat resistance (Extended Data Fig. 2E and 2F): results are not significant for NCS2. This should be reformulated.*

We rewrote the description as follows.

“Overexpression of *NCS6-oe* significantly increased the growth rate by 1.29-fold, while *NCS2-oe* increased the growth rate by 1.14-fold, although the increase was not statistically significant (Figure S6).”

*l.143 and following: it is not clear here whether all the enriched genes were tested for growth rate estimation, or specifically the 4 genes mentioned. If all were not tested, please explain better the selection of the sets of genes, in particular YBR196C-A. And what are the hypotheses regarding the other genes? Are they considered as false positives? Why? It would be nice to have a short discussion about that.*

Because we added statistical reasoning for our analysis as described above, we isolated “hits” and declared the confidence for GOFA using replicates, which we believe enough explanation for the reviewer’s concern; “*And what are the hypotheses regarding the other genes? Are they considered as false positives? Why?*”. The reason why we also analyzed *YBR196C-A* in addition to three confident GOFAs under salt stress has been included as follows.

“In addition to these three GOFAs, we also analyzed *YBR196C-A*, which was a hit only in the Pool_b-derived replicates (Figure 2A). *YBR196C-A* is recognized as an “emerging gene” and encodes an adaptive protein that localizes to the ER membrane (Figure 2B) [], while its concrete function has not been revealed yet.”

*l.194 and following: the S288C strain has a cluster of 3 ENA genes, which may explain that, ENA1 was not detected as a GOFA in this background. This could be clearly stated in the manuscript. Regarding the other selected strains, for which genome sequencing data may be publicly available, it would be nice to make a parallel between the number of copies of ENA in the genome and the detection of this gene as a GOFA.*

We included the relevant description in the Introduction section in response to the comment of Reviewer #2, added new Figure S9 to show a diagram for the *PMR* locus in these strains. The copy number of *ENA* genes of these strains were also added in Figure 3H.

*fig 3 E-G: why was not the same number of generations considered for all backgrounds?*

We added descriptions explaining about the reason as follows in Results.

“We shortened the duration of the competitive culture for CEN.PK and DBVPG6765 in comparison to BY4741, terminating it after 8 and 16 generations, respectively. This is because we consider that adaptive plasmids should already be enriched in these generations. As expected, the diversity of the pooled library, as measured by the Gini-Simpson index, decreased quickly when using CEN.PK and DBVPG6765, as opposed to BY4741 (Figure 3D).”

*l.241: “Particularly, the ENA1 and HAL genes were identified as GOFAs in CEN.PK (and*

*DBVPG6765)” this sentence can lead to confusion. ENA1 was only detected in CEN.PK and it HLA5 is the only HAL genes that was detected. Please reformulate.*

We rephrased it as follows.

“Notably, *ENA1* was only identified in CEN.PK.”

*Few random typos noticed while reading the manuscript:*

*- Extended figure1: mislabeling of the regions for which scatterplots were shown (from D to H while it should be from F to J)*

*- l.120: repetition of (Fig. 1G and 1H)*

*- fig 2D. "The cytoplasmic Ca2+ pluses" instead of "pulses"*

We are sorry for the errors. We have corrected them, and thoroughly checked our manuscript.

**Other response.**

***Have all data underlying the figures and results presented in the manuscript been provided?***

*Reviewer #1:* ***No:*** *According to the Methods section, the authors deposited DNA sequencing data and RNA-seq data in the DNA Data Bank of Japan. But it does not appear that they deposited the Oxford Nanopore sequencing data that underlies the bulk of the experimental results. It also does not appear that numerical data underlying experiments such as growth rate measurements, etc. are included as Supplementary Information.*

Nanopore data are in the process of being deposited to DDBJ, will be shown after acceptance. Numerical data are included in this submission as Supplementary Information
