# Supplementary material for: Overexpression profiling reveals cellular requirements in the context of genetic backgrounds and environments
Source: PLoS Genet. 2023 Apr 28;19(4):e1010732. doi: 10.1371/journal.pgen.1010732 (PMC10171610; doi:10.1371/journal.pgen.1010732)
Supplement: Attachment — Submitted filename: Resposes_to_reviewer_comments.docx [file pgen.1010732.s027.docx]

We once again express our gratitude for the constructive comments from the reviewer. Below, we describe our response to the reviewer’s comments. We have made revisions to the paper in response to all reviewer comments.

*1) I agree that performing a statistical test for whether or not a given strain changed in abundance over the course of a multi-generational co-competition is bound to return positive hits for essentially every strain when comparing to the original abundances. Still, I appreciate that you applied an FDR threshold. Rather, the statistical analysis that is more important to perform is whether the hits from one replicate of an experiment were obtained more often than expected by chance in the other replicates. Off the top of my head, I am not certain how this is performed with more than two replicate experiments, but with two replicates people often use hypergeometric tests. From your Venn diagrams it certainly seems that the degree of overlap is far more than expected by chance, and so if you do the statistical tests I think they are highly likely to return positive results. But, for instance, the statement, “Seventeen genes in CEN.PK and 13 genes in DBVPG6765 were likely to be GOFAs under salt stress, as they were identified as hits in all the triplicate experiments,” is not correct as stated; the 17 genes are likely to be GOFAs because they were identified in all the triplicate experiments AND there were very few hits identified in just one or two replicates, that is, there is much more overlap in hits than expected by chance.*

We performed permutation tests to assess the significance of whether the number of genes hit by replicates was greater than chance. The permutation test allows us to evaluate the significance of the observed overlaps without making assumptions about the distribution of the data. For instance, our analysis of the CEN.PK case has shown that the observed overlaps of genes being hit in 2/3 and 3/3 replicates were statistically significant, indicating a non-random pattern in the distribution of gene hits across the replicates. However, the overlap of genes being hit in 1/3 replicates was not found to be significant.

Based on these findings, we have revised the statement in our manuscript as follows: "Seventeen genes in CEN.PK and 13 genes in DBVPG6765 were hit in all the triplicate experiments. The permutation test demonstrated that the observed overlaps of genes being hit in 2/3 and 3/3 replicates were statistically significant (CEN.PK hitin 2/3 *p* < 0.0001, hit in 3/3 *p* < 0.0001, and DBVPG6765 hit in 2/3 *p* < 0.0001, hit in 3/3 *p* < 0.0001), indicating a non-random pattern in the distribution of gene hits across the replicates. However, the overlap of genes being hit in 1/3 replicates was not found to be significant (CEN.PK hit in 1/3 *p* = 1.00 and DBVPG6765 hit in 1/3 *p* *=* 1.00). These findings support the claim that the overlapped genes are likely to be GOFAs. "

We have also added a description of how we performed the test in the Materials and Methods section.

*2) The revised introduction includes more background about overexpression profiling, which is welcome. You cited three examples of recent overexpression profiling screens (citations 20-22); to this list I think you should add Arita et al. Mol Syst Biol 2021, Magtanog et al. Nat Biotech 2011, and Payen et al. PLoS Genet 2016 (and perhaps others). In particular, Payen studied overexpression mutants that increased in abundance, and thus statements like, “However, those libraries have never been used to isolate beneficial genes” are incorrect. It would also be appropriate to mention that Robinson et al. tested the effects of overexpression in multiple strain backgrounds (lines 96-97).*

We cited these works and mentioned Payen’s work instead of “never.” Moreover, we also mentioned Robinson’s work as focusing on multiple strain backgrounds.

*3) I like the linear scatterplot visualization to showing the hits from the overexpression profiling. However, the x-axis is not labelled, and I cannot tell what the x-axis should be, such as in figure 1D-H.*

We added the x-axis labels to the linear scatterplots in Fig. 1, 3, 4, 6, and S15, and explanation of the labels in their legends.

*4) If I understand correctly, pool_a and pool_b are replicate pools generated from the same arrayed strains generated in Makanae et al.? If that’s right, I would not refer to them as “independent” as on line 137, as any mutation, etc. present in the Makanae arrayed strains will be present in both pool_ a and pool_b. You say in line 155-157 that “if the same overexpression strain is enriched after multiple independent cultures, it strongly suggests that the overexpression strain was adaptive rather than resulting from mutation.” This would be true if the pools were truly independent, but as mentioned above, if the pools involve strains that had a common progenitor, any mutations experienced by those progenitors will be shared in the pools. I do not think you need to redo the experiments or analyses due to this concern, but it would be best to acknowledge it, either in the Results or the Discussion. The fact that you obtain hits from related but independent overexpression strains (such as NCS6 and NCS2) gives confidence that the results are not predominantly arising from shared secondary mutations.*

In Makanae et al. we actually constructed two “independent” sets of overexpression libraries, namely isolated two independent colonies of plasmid transformants (Figure 1), indicating that the two pools do not share the same mutations. Of course, if BY4741 we used in our lab has specific mutants, they are shared. Our pool_a and pool_b were created by pooling each set. In the “Result” section, we emphasized that two independent overexpression array strains were generated in Makanae et al.

Figure 1. From ttps://genome.cshlp.org/content/23/2/300/F1.large.jpg, Makanae et al., 2013.

*There are still a lot of language issues that will need to be addressed. Here are some that I caught:*

*1) Line 96: “Previous” should have a lowercase “p.”*

*2) Line 137: You say the overexpression pools were maintained as a “liquid stock in a 96-well format”: do you mean as a frozen stock?*

*3) Line 178: When you say “as expected, the dosage sensitive genes identified in our previous studies […] were omitted,” I think “omitted” may be being misused in this context. I would interpret “omitted” to mean you removed them from consideration. Do you mean that those genes declined in abundance?*

*4) Line 201: Probably you should specify that the growth rate was increased by 1.29-fold in 40C, if that is what you meant.*

*5) Line 255: Remove the comma after “ENA1.”*

*6) Line 320: I think you’re missing a word after “were no longer.”*

*7) Line 322: I think you want to remove the word “under.”*

*8) Line 333: Hyphenate “salt-tolerant.”*

*9) Line 466: Overexpression profiling is not a new experimental system.*

*10) Line 585: I think you’re missing a word after “pooled libraries were.”*

*11) Line 590: Capitalize “the.”*

*12) Line 594: I don’t understand the sentence that begins “The mixed PCR products of and introduced into […]”. 13)*

*Line 599: I think you mean “plates” instead of “templates.”*

We apologize for these errors and greatly appreciate your careful review of them. We have corrected these errors based on the suggestions.

*Reviewer #1:* ***No:*** *The authors say that large-scale datasets are in the process of being deposited, which is good, but it would appear they have not yet been deposited.*

We deposited those datasets in the DNA Data Bank of Japan (accession number: DRA015709) and added the below sentence to “Overexpression profiling” in the method section. “The raw data were available in the DNA Data Bank of Japan (accession number: DRA015709)”.
